# Prevalence and policy of occupational violence against oral healthcare workers: systematic review and meta-analysis

**DOI:** 10.1186/s12903-019-0974-3

**Published:** 2019-12-12

**Authors:** Nada O. Binmadi, Jazia A. Alblowi

**Affiliations:** 1 Oral Diagnostic Department, Faculty of Dentistry, King Abdulaziz University, Jeddah, Saudi Arabia; 2 Periodontology Department, Faculty of Dentistry, King Abdulaziz University, Jeddah, Saudi Arabia

**Keywords:** Workplace violence, Oral healthcare workers, Meta-analysis, Verbal abuse, Sexual harassment, Aggression

## Abstract

**Background:**

Occupational violence is considered unlawful in professional environments worldwide. In the healthcare industry, including dentistry, the safety of workers is essential, and it is of the utmost importance to ensure patient and employee safety and provide quality care. This study aimed to evaluate the prevalence of violence and associated workplace policies among oral healthcare professionals. Additionally, it aimed to identify the factors associated with violence and their impact on oral healthcare workers.

**Methods:**

A systematic review and analysis of the literature was conducted using PubMed, ScienceDirect, Scopus, Web of Science, Cochrane Library and ProQuest. Original articles written in English and published between January 1992 and August 2019 were included in the analysis.

**Results:**

A total of 980 articles were found, and eight were selected for analysis. The violence experienced by healthcare workers included both physical and non-physical forms, such as shouting, bullying, and threatening; it also included sexual harassment. The impact of violence on workers manifested as impaired quality of work, psychological problems, and, although rare, quitting the job. With regard to dental healthcare, awareness of occupational violence policies among dental professionals has not been previously reported in the literature.

**Conclusions:**

The increasing incidence of occupational violence against oral healthcare workers indicates the need for the implementation of better protective measures to create a safe working environment for dental professionals. There is a current need for increasing awareness of workplace violence policies and for the detection and reporting of aggression and violence at dental facilities.

## Background

With the increasing incidence of violence worldwide, occupational violence has become a reality for all kinds of workers in various kinds of workplaces. Violence in the workplace is the third leading cause of death, and it leads serious safety and health issues that undermine the ability of health workers to focus on their jobs [1]. According to the World Health Organization (WHO), violence in the healthcare industry negatively influences not only employees but also the workplace ambiance, colleagues, employers, families, and society as a whole, and it may result in injury, death, or psychological harm to the affected person. A WHO study reported that this type of violence could reduce health services for the general population, with a consequent increase in healthcare costs. Moreover, it leads to monetary losses ranging in the hundreds of dollars as well as to the loss of workdays [2].

Dentistry is more susceptible than other healthcare areas to occupational violence in hospitals and clinics because dental clinics are usually crowded with patients. The workers at dental healthcare facilities are exposed to threats to personal health during their duties in addition to the biohazard dangers they face due to the use of sharp instruments and chemical components [3, 4]. Oral healthcare occupational violence can be categorised into various forms, such as verbal abuse, property damage or theft, physical abuse, sexual harassment, and bullying. For example, a study performed in Nigeria showed that more than 70% of dental staff experienced verbal assault, although physical violence was reported to be rare [1]. Other studies have focused on sexual harassment due to the major increase in the number of women as healthcare professionals [5–7]. The most common perpetrators are patients and their relatives [1, 5]. Several factors contribute to violence against dental professionals, including long waiting times, the cancellation of appointments, the outcomes of patient treatment, alcohol intoxication, psychiatric patients, and expensive bills [1]. Additionally, the paucity of manpower to handle the increasing number of patients at dental healthcare centres and the uneven geographic distribution of these centres worsen the situation. Because of the aesthetic value associated with the face, any error on the part of a dentist can trigger rage and anger among patients, consequently leading to violent incidents [8].

A consensus definition of healthcare workplace violence, such as bullying, verbal abuse, sexual harassment, threat and physical abuse, was agreed upon and proposed in 2016 by Boyle and Wallis [9]. The absence of information on the prevalence and policies associated with workplace violence among dental professionals is related to poorly defined implementation and solutions. The current situation demands a comprehensive programme aimed at preventing workplace violence [10]. The current systematic review and meta-analysis are meant to evaluate the prevalence of violence experienced by oral healthcare professionals and the associated workplace policies at their facilities. Furthermore, the present study also aims to identify the factors associated with violence and their impact on oral healthcare workers.

## Methods

We report this manuscript in accordance with the Preferred Reporting Items for Systematic Reviews and Meta-analysis (PRISMA statement) guideline [11]. All methods used in this review were conducted in strict accordance with the Cochrane Handbook for Systematic Reviews of Interventions [12].

### Study design

The present systematic literature review search was performed using the systematic literature review tool Parsifal (https://parsif.al/). It is an easy-to-use web-based tool for designing protocols and extracting and managing data. Six databases were searched, specifically MEDLINE (PubMed), ABI/INFORM (ProQuest), Scopus, Web of Science, Cochrane Library, and ScienceDirect (Table 1).

**Table 1:** Search terms for each database

Database	Search Term	Results
PubMed	((dentist OR Oral healthcare workers OR dental professionals OR dental assistant OR dental hygienists) AND (violence OR bullying OR threats OR harassment) NOT (child Abuse))	384
Cochrane Library	((dentist OR Oral healthcare workers OR dental professionals OR dental assistant OR dental hygienists) AND (violence OR bullying OR threats OR harassment))	10
Scopus	((dentist OR Oral healthcare workers OR dental professionals OR dental assistant OR dental hygienists) AND (violence OR bullying OR threats OR harassment))	72
Science Direct	(((dentist OR dental assistant OR dental hygienists) AND (violence OR bullying OR harassment)) AND (Cross-section OR Cross-sectional) NOT (Child Abuse)))	447
Web of Science	((dentist OR Oral healthcare workers OR dental professionals OR dental assistant OR dental hygienists) AND (violence OR bullying OR threats OR harassment) NOT (child Abuse))	44
ProQuest	((dentist OR Oral healthcare workers OR dental professionals OR dental assistant OR dental hygienists) AND (violence OR bullying OR threats OR harassment))	23

### Search strategy

**Fig. 1:**
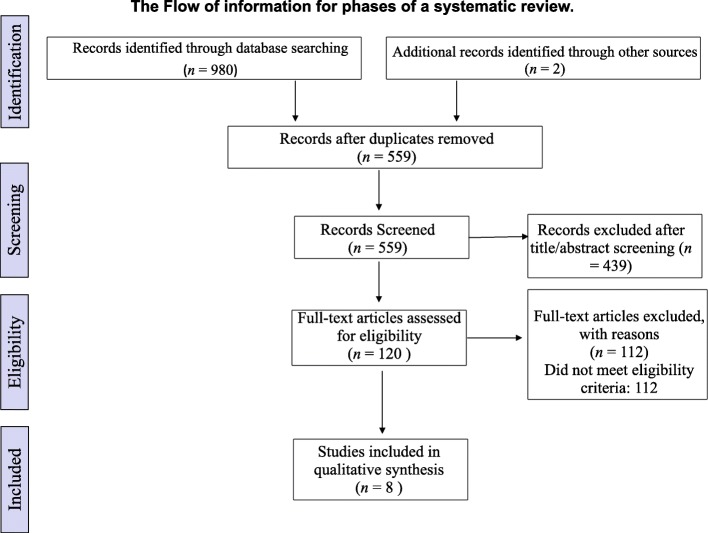
Flow of information through different phases of a systematic review

The keywords employed in the search strategy included “dentist” OR “oral healthcare workers” OR “dental professionals” OR “dental assistant” OR “dental hygienists” OR “general practitioners” AND “violence” OR “bullying” OR “threats” OR “harassment.” Only peer-reviewed articles that were written in English were considered. The final sample consisted of eight articles published between 1992 and 2019. After excluding duplicates, non-English articles, and irrelevant articles, the authors systematically reviewed the articles that met the predetermined criteria and studied only oral healthcare professionals’ knowledge related to detecting aggression, reporting violence, the prevalence of violence, and the awareness of occupational healthcare policies among dental professionals following the (PRISMA) guidelines [11], as shown in Fig. 1.

### Study selection process and eligibility criteria

The authors screened the titles and abstracts of the retrieved literature records. For titles and abstracts that were deemed relevant to the research question, the full-text articles were obtained and screened for eligibility according to the following criteria: 1) studies whose population was oral healthcare workers, interns, or students, 2) studies that assessed the prevalence of violence and associated workplace policies among oral healthcare professionals, 3) studies reporting any information about the causes of this violence, and 4) observational prospective or retrospective studies.

Articles were also excluded if they included a discussion of violence among general practitioners that included dentists as part of the team.

### Data extraction

The data extracted included the year of publication, author’s name, country, study type, population, duration of study, sample size, response rate, demographic data (gender and age), type of violence, number of violent incidents per population, risk, effect, policy, action taken, percentage of each gender affected, and the persons responsible for the mistreatment.

### Quality assessment

The Newcastle-Ottawa Scale (NOS) was used to evaluate the quality of each included study. It includes eight assessment items for quality appraisal, including ‘selection’, ‘comparability’ and ‘outcome’. According to the NOS scoring standard, cross-sectional studies can be classified as low quality (scores of 0–4), moderate quality (scores of 5–6) and high quality (scores ≥7), Additional file 1.

### Statistical meta-analysis

A random effects model meta-analysis was performed to evaluate the pooled success rate. Publication bias, fail-safe N, Begg and Mazumdar rank correlation, and the heterogeneity of the studies were determined using Michael Borenstein’s Comprehensive Meta-Analysis (CMA) programme, version 3 (Englewood, NJ, United States).

## Results

In total, 980 articles were found based on the search strategy. Of these articles, eight were included after the removal of duplicates and non-English studies; these articles are summarised in Table 2. According to the NOS tool for quality assessment, four studies showed high quality (≥ 8 points), and four showed moderate quality (5 and 6 points), Table 3. The studies were cross-sectional surveys from Brazil, Nigeria, Sri Lanka, the United States of America, Pakistan, and the United Kingdom. Five of these studies were conducted at a dental school, one was conducted at a postgraduate dental hospital, and two were conducted at dental offices. Most studies assessed violence over a period of 2 to 12 months. The mean age ranged from 22 [5, 14] to over 60 years [15]. In most cases, male patients or coworkers were responsible for the mistreatment or violence. Sexual harassment was predominant among the types of violence that occurred in the field of dentistry, with a prevalence rate ranging from 6.8 to 54% in almost all the studies [1, 7]. Verbal abuse included shouting; extremely loud shouting was also frequently reported, and its prevalence ranged from 8.2 to 58.7% [13, 14]. Another type of violence reported was bullying, with a prevalence rate of 22 to 36.8% [1, 16]. Physical abuse was the least frequently reported type of violence, with a prevalence rate ranging from 4.6 to 22% [13, 14].

**Table 2:** Characteristics of the included studies focusing on violence among oral healthcare workers

Author, country, year	Study duration (months)	Sample	Sample size	Response rate (%)	Percentage of violence (%)	Demographics: Age (years) and sex	Type of violence	Action and policy
Garvin and Sledge, USA, 1992 [6]	2	Dental hygienists	650	72.6	26.3	Age mean = 28.9, 100% females	Sexual harassment	Action: terminated employment, legal action, no action, reported incident. No policy
Pennington et al., USA, 2000 [7]	NA	Dental hygienists	540	53	54	Mean of age 40, 99% females	Sexual harassment	Action: File formal complaints. No policy
Garbin et al., Brazil, 2009 [5]	NA	Dental students	254	82	15	Age range:19–32 years (mean = 22), 65.9% females and 34.1% males	Sexual harassment (15%)	Action: Inform the mentor or do nothing. No policy
Steadman et al., United Kingdom, 2009 [13]	12	Postgraduate hospital dentists	227	60	25	NA	Bullying (25%), threat (8.2%), verbal abuse (8.2%), sexual harassment (7.5%), property damage (1.5%)	Action: Inform the mentor. No policy
Azodo et al., Nigeria, 2011 [1]	12	Dental professionals	175	78.9	31.9	NA	Bullying and physical abuse (22%), loud shouting (50%), sexual harassment (6.8%), threat (22.7%)	Action: NA. No policy
Premadasa et al., Sri Lanka, 2011 [14]	9	Junior dental students	72	91	50	Age range: 20–23, 67.7% females and 32.3% males	Loud shouting (50%), physical abuse (4.6%), sexual harassment (11.5%), threat, verbal abuse (58.7%)	Action: Do nothing, inform friend or family, inform the mentor. No policy
McCombs et al., USA, 2018 **[**15**]**	6	Dental hygienists	240	64	24	Age range: 20 – > 60, 97% females and 3% males	Workplace bullying, opinions and views ignoring, and unmanageable workloads	Action: NA. No policy
Ullah et al., Pakistan, 2018 [16]	6	Dental interns	135	92.59	36.8%	Mean age 24.0 ± 1.3, 69.6% females and 30.4% males	Workplace bullying, unmanageable workload, and being ignored or excluded	Action: Only 14.5% reported a complaint. No policy

**Table 3:** Quality assessment of the enrolled studies

Study	Selection				Comparability	Outcome		Results
	Representativeness of the sample	Sample size	Non-respondents	Ascertainment of the exposure (risk factor):	Comparability of subjects in different outcome groups on the basis of design or analysis	Assessment of outcome	Statistical test	
Garvin and Sledge [6]	*	*		*		*	*	5/10
Pennington et al. [7]	*	*	*	*		*	*	6/10
Garbin et al. [5]	*	*		*	**	**	*	8/10
Steadman et al. [13]	*	*		*	**	**	*	8/10
Azodo et al. [1]	*	*		*		*	*	5/10
Premadasa et al. [14]	*	*	*	*	**	**	*	9/10
McCombs et al. [15]	*	*		*		**	*	6/10
Ullah et al. [16]	*	*		*	**	**	*	8/10

Scale of studies (points): Very Good: 9–10, Good: 7–8, Satisfactory: 5–6, and Unsatisfactory: 0 to 4

A higher proportion of females than males was exposed to aggression and violence among oral healthcare workers, as reported by Garvin and Sledge [6], Pennington et al. [7] and Premadasa et al. [14]. Only one study reported a male predominance in exposure to violence [5]. The gender difference was statistically insignificant in the other studies [1, 6].

Several studies have discussed other factors associated with violence against oral healthcare workers. Long waiting times and a lack of staff training were the most frequent factors reported [1, 5]. Other contributing factors included alcohol intoxication and psychiatric illness, which accounted for 9.1 and 4.5% of the variables, respectively [1]. In all of the studies, the targets of violence reported negative effects of the abuse, such as a decline in ethical values, but few reported psychological stress or any impairment in their performance.

Most studies concluded that healthcare workers did not take any action to deal with the violence or abuse inflicted upon them. However, some abused workers discussed the issue with family members or close friends and reported it to their manager, mentor, or administrative office [5, 13, 14]. Ullah et al. [16] discussed the important reasons that those who experienced bullying did not complain: the majority of respondents (28.8%) thought that complaining was useless; 22% were afraid of the consequences, especially when the perpetrator was a senior faculty member; 20.8% of them felt they could deal with incidents on their own; and 16.9% considered the incident not sufficiently serious. This reflects the importance of educational and instructional courses on how to address such situations.

The fail-safe N was 453, and Kendall’s tau *ß* was 0.179, with a one-tailed *p* = 0.268. The outcomes of both of these tests indicated a lack of publication bias. The heterogeneity assessment reported a Q-value of 172.423, with d_f_ = 7, *p* < 0.001 and *I*^2^ = 95.94 (95% confidence interval: 0.321 to 0.362), suggesting that approximately 96% of the observed variance in the effects was real or heterogeneous.

**Fig. 2:**
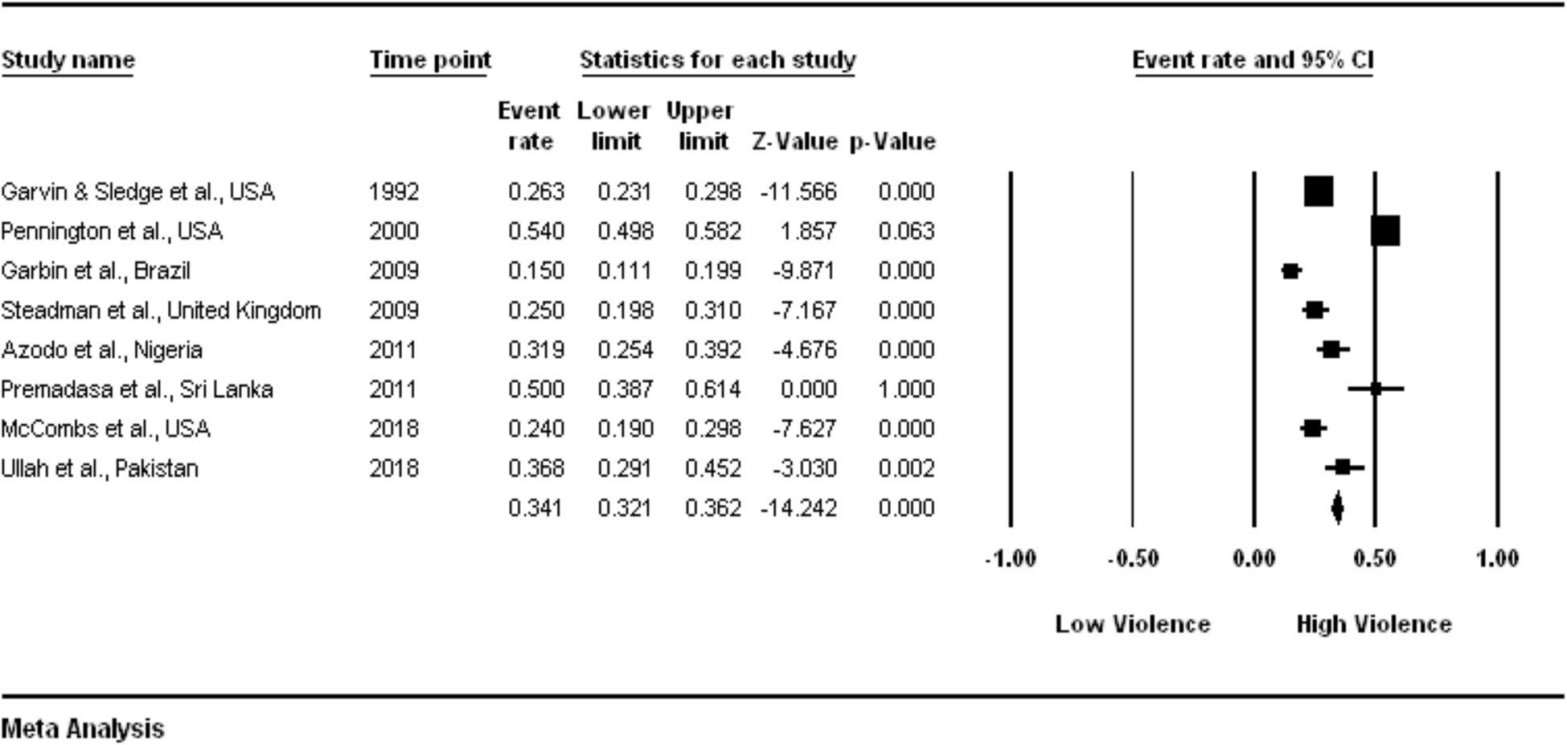
Detailed statistics for each study, with event rates calculated using random-effects model meta-analysis

The pooled estimate for the random-effect model was 0.320 (Fig. 2). An examination of the plot showed the following results:

- The violent events were noticeably diverse from study to study;
- The violent events ranged from a low of 15.0% to a high of 54.0%; and
- The mean prevalence was 0.341 (32%), with a CI ranging from 0.321 to 0.362.

## Discussion

In practice and in the literature, the terms “aggression” and “violence” are often used interchangeably. Therefore, it is very important to define each term to solve this confusion. Human aggression was defined by Hills and Joyce [17] as “any action or behaviour directed by a perpetrator towards a target that is characterised by the perpetrator’s intention of causing harm or damage for the purpose of achieving a proximal or distal outcome, the target’s motivation to avoid the action or behaviour and the violation of norms that the action or behaviour represents.” On the other hand, workplace violence is a specific term that refers to incidents in which employees are harassed, endangered or attacked in work-related conditions, causing an explicit or implicit threat to their security, or health or well-being [18]. In the psychological and sociological literature, the phrase ‘workplace aggression’ is used most often, while ‘workplace violence’ is more frequently found in the literature on industrial and health professions [19].

Occupational violence, in any form, should not be acceptable, irrespective of the frequency of its occurrence. Personal safety should be a priority in any professional environment. Professionals have consistently underestimated the prevalence of violence among healthcare workers in general, especially in dental healthcare centres. The occurrence of occupational violence demonstrates the need for improved protection measures to create a safe working environment for dental professionals [1]. Several systematic reviews have been conducted in settings other than dental professional centres, such as emergency departments and nursing settings [20, 21]. A study among National Health System (NHS) staff showed that dentists were the group that least frequently reported work aggression or violence compared to emergency, acute care, and mental departments [3]. To the best of our knowledge, this is the first review conducted on violence against oral healthcare workers.

Violence in the workplace is associated with various negative consequences. First, it results in both physical and mental trauma for the victims. The victims find it difficult to concentrate on their jobs, as manifested by a decreased interest in work, increased leaves of absence, and decreased job satisfaction. Worker compensation losses, decreased productivity, and the deterioration of ethical values are additional outcomes. Most of the time, the incidence of violence goes unnoticed or underreported [2]. In the studies reviewed in this paper, the victims reported experiencing psychological stress and impairment to their jobs.

The perpetrators of physical violence are usually people from outside the office, for example, patients and their relatives, whereas verbal abuse and bullying are usually carried out by senior colleagues [22]. This finding is inconsistent with a study by Premadasa et al. [14], which found that senior students and instructors are responsible for most sexual harassment incidents. We found that women working in dentistry reported a high prevalence of occupational violence compared to men, a finding similar to several previous studies in other healthcare areas [23, 24]. The availability of limited information in some studies prevented us from calculating the number of males and females who had undergone violent experiences. The mean age of the participants in the studies demonstrated that most of the people exposed to violence were young, a finding that was consistent with many studies [23, 25]. The review articles discussed various types of occupational violence, among which sexual harassment was the most frequently mentioned in all studies, although it was not the most frequently occurring type of incident. Verbal abuse was the most frequently occurring type of violent incident reported in two studies [1, 14]. These findings are comparable to other studies conducted in India and the United Kingdom [26, 27].

Dental professionals usually take no action against abuse due to perpetrator-target relationships [28]. There is clearly a lack of institutional justice and standards or guidelines with regard to dealing with the issue. No intervention studies in the dentistry field have investigated the role of awareness and education in improving the detection and reporting of abuse [29]. A new strategy should be designed to mitigate violence and abuse against dentists and their staff that involves a combination of individual, organisational, social, and political support.

No incidence of violence should remain unnoticed or underreported. However, most incidences of violence are overlooked, which results in worker dissatisfaction in the long run. Addressing the causes leading to violence at dental care centres and improving the quality of service provided may help reduce the likelihood of violent incidents. Improving treatment and reducing waiting times could strengthen doctor-patient communication. Effective interventions in terms of the enhancement of security at dental healthcare centres and sufficient social support should be implemented. Several studies highlighted the importance of social support in reducing anger, frustration and conflict in the work environment in addition to allowing employees to voice their experiences without any concerns about repercussions [30, 31].

At the organisational level, healthcare managers and policy makers should shoulder the responsibility for planning and implementing appropriate guidelines and interventions for reporting and preventing incidences of violence [32]. Organisations should also support the development and analysis of interventions to enhance professional detection and reporting of abuse, including staff documentation, documentation by the attacked employee, contact by legal representatives, contact by outside medical facilities, security patrols, camera installation, the use of bright lights at night, staff support, and truthful media reporting [33]. Additionally, the existence of a violence prevention programme in the workplace may prove beneficial in combating and eliminating any violence-related risk. Individuals in higher positions within the hierarchy should act and respond promptly to any incident of physical or verbal violence, thereby effectively assuring workers’ safety and protection.

The low priority society places on the rights and safety of healthcare providers is another contributing factor. Therefore, dental organisations and societies, such as the American Dental Association (ADA) [34], should act to protect the profession and advocate for policies that prevent violence against dentists and dental staff. Following the Occupational Safety and Health Administration (OSHA), oral healthcare workers are encouraged to attend training that raises their awareness about the risks of aggression and violence; however, these steps are still insufficient to prevent the issue [10]. Similarly, the government should enact strict laws and regulations that require the investigation of any incidence of violence in dental healthcare centres with the utmost transparency. Abiding by such legislation should be mandatory for all professional centres. Additional steps to curb violence in the workplace may involve conducting a regular questionnaire-based survey to assess awareness among health professionals while keeping their identity secret. Several policies need to be adopted to reduce violent incidents and protect dental professionals. In cases of abuse and violence by patients, all healthcare services should be denied. For example, in England, after violence against any employee by a patient is reported, the patient is removed from the NHS and cannot schedule an appointment with any dentist registered with the NHS [35]. Anti-violence and anti-harassment groups for dental professionals should act in pursuit of a healthy and safe working environment.

The present systematic review and meta-analysis quantified the prevalence of violence towards dental professionals. The findings were limited because of the paucity of articles related to this issue that discussed the incidence of violence against oral healthcare workers and the actions taken to prevent it. The statistical analysis showed that there was no publication bias among the assessed articles. However, it should be noted that these results do not necessarily reflect the significance of the results due to the limited number of studies and their statistical power.

## Conclusions

Violence of various forms is a common occurrence in the workplace. The present systematic review demonstrated a moderate prevalence of violence at dental healthcare centres, especially against females. The most commonly observed violence type was sexual harassment, followed by verbal and physical abuse. Healthcare workers at dental centres should inculcate a zero-tolerance approach and should not ignore any warning signs. The lack of implementation of strict guidelines at these centres augments the situation. There is a current need to design and adopt improved policies through collaborations between dental organisations and societies and law-making bodies. Further high-quality studies are needed to investigate the association between violence against dental healthcare workers and the related factors.

## Supplementary information

**Additional file 1.** Quality Assessment Scale for cohort studies adapted from the Newcastle-Ottawa scale.

## Abbreviations

ADA: American Dental Association
CMA: Comprehensive Meta-Analysis
NHS: National Health Service
NOS: Newcastle-Ottawa Scale
OSHA: Occupational Safety and Health Administration
PRISMA: Preferred Reporting Items for Systematic Reviews and Meta-Analyses
WHO: World Health Organisation
